# Parental Knowledge and Practices of Sleep Hygiene Among Children in Saudi Arabia: A Cross-Sectional Study

**DOI:** 10.7759/cureus.64292

**Published:** 2024-07-10

**Authors:** Amani Alharbi, Maryam Bajaifar

**Affiliations:** 1 Pediatrics, Al-Azzizyah Children Hospital, Jeddah, SAU

**Keywords:** educational interventions, pediatric care, cross-sectional study, saudi arabia, children's health, parental practices, parental knowledge, sleep hygiene

## Abstract

Background: Sleep hygiene is crucial for child development, influencing physical health, cognitive function, and emotional well-being. Parental knowledge and practices significantly influence children’s sleep habits, yet gaps in understanding persist, impacting sleep quality and overall health outcomes. In Saudi Arabia, rapid societal changes and modern lifestyles pose unique challenges to maintaining healthy sleep habits among children. This study aims to assess parental knowledge and management of sleep hygiene, providing insights for targeted interventions tailored to Saudi cultural contexts.

Methods: This cross-sectional study assessed parental knowledge and management of sleep hygiene among children in Saudi Arabia. Participants (N=729) were recruited from pediatric clinics and online forums, comprising parents with at least one child aged 0-18 years who completed surveys in Arabic or English. A comprehensive survey collected demographic data, parental sleep hygiene knowledge, practices, and concerns. Data were gathered between January and March 2024 via online and clinic-based distribution and analyzed using SPSS version 25 for descriptive statistics.

Results: The survey was completed by 729 participants, predominantly aged 25-44 years (70.4%), holding predominantly bachelor’s degrees (34.7%), and employed full-time (49.7%). The majority reported having 2-3 children (54.9%). Findings indicated that 69.1% (504 participants) correctly identified school-aged children’s sleep needs, and 71.0% (518 participants) recognized the importance of limiting electronic device use before bedtime. Sleep management practices revealed that 81.3% (592 participants) of parents adhered to bedtime routines, and 65.6% (478 participants) managed electronic device use appropriately. Bedtimes typically ranged from 7 to 9 PM for 90.5% (658 participants) of children, with wake-up times clustered between 6 and 8 AM for 75.6% (551 participants). Parental concerns showed reliance on online resources (60.4%) and pediatricians (54.7%) for sleep information, with 73.9% (539 participants) expressing interest in further education on sleep hygiene.

Conclusions: This study highlights parental awareness of sleep hygiene practices in Saudi Arabia but underscores gaps in knowledge regarding caffeine effects and optimal napping practices. Tailored educational interventions are essential to enhance parental understanding and promote healthier sleep habits, thereby optimizing child well-being in the region.

## Introduction

Sleep is a fundamental component of child development, influencing physical growth, cognitive function, and emotional regulation [1]. Adequate and quality sleep during childhood is critical for overall health and well-being, with long-term implications extending into adulthood [2]. Despite its importance, sleep disturbances and inadequate sleep are common among children worldwide, including those in Saudi Arabia [3]. These issues can lead to adverse outcomes such as impaired academic performance, behavioral problems, and increased risk of chronic health conditions [4].

Parental knowledge and practices play a crucial role in shaping children’s sleep habits [5]. Parents who are well-informed about sleep hygiene can implement effective strategies to promote healthy sleep patterns in their children [6]. Sleep hygiene encompasses a range of practices and environmental factors that contribute to good quality sleep, including consistent bedtimes, limiting electronic device use before bed, and creating a conducive sleep environment [7].

Previous studies have highlighted gaps in parental knowledge regarding sleep needs and optimal sleep practices for children [8]. These gaps can result in suboptimal sleep management practices, exacerbating sleep problems among children. Given the cultural, social, and environmental differences across regions, it is essential to explore parental knowledge and sleep management practices within specific populations [9].

In Saudi Arabia, rapid modernization and lifestyle changes have introduced new challenges to maintaining healthy sleep habits among children. Increased use of electronic devices, changes in dietary patterns, and shifts in daily routines may contribute to sleep disturbances [10]. Understanding the current state of parental knowledge and practices regarding sleep hygiene in Saudi Arabia is critical for developing targeted interventions and educational programs.

This study aims to assess parental knowledge and management of sleep hygiene in children in Saudi Arabia. By identifying common practices, knowledge gaps, and parental concerns, this research seeks to inform the development of effective strategies to improve sleep health among children in this region.

## Materials and methods

Study design and participants

This cross-sectional study was conducted to assess parental knowledge and management of sleep hygiene in children. Parents residing in Saudi Arabia were recruited through pediatric clinics and online parenting forums. Participants were eligible if they had at least one child aged 0-18 years and were able to complete the survey in Arabic or English. Informed consent was obtained from all participants prior to their inclusion in the study.

Survey instrument

A comprehensive survey was developed to collect data on demographic characteristics, parental knowledge of sleep hygiene, sleep management practices, and parental concerns regarding sleep hygiene. The survey consisted of four sections: Demographic information, which included questions on the participant’s age, education level, employment status, and the number and age range of their children; knowledge of sleep hygiene, which assessed parents’ knowledge about the recommended hours of sleep for school-aged children, practices for good sleep hygiene, and the ideal time for children to stop using electronic devices before bedtime, as well as parental understanding of recommended practices for promoting good sleep and the ideal age for children to stop napping during the day; sleep management practices, which gathered information on parents’ implementation of regular bedtime routines, their children’s use of electronic devices before bedtime, typical bedtimes and wake-up times, frequency of nighttime awakenings, difficulty falling asleep, and strategies used to help children fall asleep, including questions on how parents handled bedtime resistance; and parental concerns and education, which explored parents’ perceptions of their knowledge about sleep hygiene, sources of information, and interest in receiving further education on sleep hygiene for children (Appendices).

Data collection

The survey was distributed online and in-person between January and March 2024. For the online distribution, links to the survey were shared on popular parenting forums and social media groups. Paper surveys were distributed in pediatric clinics to ensure the inclusivity of participants without internet access. The survey was available in both Arabic and English to accommodate the diverse linguistic preferences of the participants.

Data analysis

Data were analyzed using descriptive statistics to summarize the demographic characteristics, parental knowledge, sleep management practices, and concerns regarding sleep hygiene. Frequencies and percentages were calculated for categorical variables. All data analyses were performed using SPSS version 25 (IBM Corp., Armonk, NY).

## Results

Demographic characteristics of participants

A total of 729 parents participated in the study. The majority were aged between 35 and 44 years (289 participants, 39.7%), followed by those aged 25-34 years (224 participants, 30.7%). In terms of education, 253 participants (34.7%) held a bachelor’s degree, while 154 participants (21.1%) had secondary education. Most participants were employed full-time (362 participants, 49.7%) and had two or three children (185 participants, 25.4%, and 216 participants, 29.6%, respectively) (Table 1).

**Table 1:** Demographic characteristics of participants (N=729) Data are presented as frequency (n) and percentages (%).

Characteristic		Frequency (n)	Percentage (%)
Age (years)	Under 25	72	9.9
	25-34	224	30.7
	35-44	289	39.7
	45-54	111	15.2
	55 and above	33	4.5
Education level	Primary education	41	5.6
	Secondary education	154	21.1
	Some college	144	19.7
	Bachelor’s degree	253	34.7
	Master’s degree	104	14.3
	Doctorate or equivalent	33	4.5
Employment status	Employed full-time	362	49.7
	Employed part-time	76	10.4
	Self-employed	74	10.1
	Unemployed	78	10.7
	Homemaker	115	15.8
	Retired	16	2.2
	Student	8	1.1
Number of children	1	68	9.3
	2	185	25.4
	3	216	29.6
	4	141	19.3
	5 or more	119	16.3
Age range of children	0-2 years	258	35.4
	3-5 years	349	47.9
	6-12 years	511	70.1
	13-18 years	264	36.2

Parental knowledge of sleep hygiene

Most parents (504 participants, 69.1%) correctly identified that school-aged children need 10-11 hours of sleep per night. Additionally, 533 participants (73.1%) recognized that watching TV before bed is not recommended. When asked about the ideal time for children to stop using electronic devices before bedtime, 518 participants (71.0%) correctly indicated that it should be 1 hour before bed. A large majority (654 participants, 89.7%) correctly identified that a dark and quiet bedroom is recommended for good sleep. Lastly, 470 participants (64.5%) correctly indicated that children should stop napping by age 4-5 years (Table 2).

**Table 2:** Parental knowledge of sleep hygiene (N=729) Data are presented as frequency (n) and percentages (%).

Question	Correct Answer	Frequency (n)	Percentage (%)
How many hours of sleep do school-aged children (6-12 years) typically need?	10-11 hours	504	69.1
Which of the following is NOT recommended for good sleep hygiene in children?	Watching TV before bed	533	73.1
Ideal time to stop using electronic devices before bedtime?	1 hour before bed	518	71.0
Recommended practice for promoting good sleep?	Dark and quiet bedroom	654	89.7
Age children should ideally stop napping?	4-5 years	470	64.5

Sleep management practices

Over half of the parents (372 participants, 51.0%) reported always having a regular bedtime routine, while 221 participants (30.3%) reported having a routine most of the time. Regarding electronic device use before bedtime, 251 participants (34.4%) reported their children sometimes used devices in the hour before bed, and 142 participants (19.5%) reported often.

Typical bedtimes for children on school nights were between 8 and 9 PM for 296 participants (40.6%), with wake-up times between 7 and 8 AM for 290 participants (39.8%). Nighttime awakenings were reported never by 287 participants (39.4%), and rarely by 248 participants (34.0%). Difficulty falling asleep was reported never by 283 participants (38.8%), and rarely by 257 participants (35.3%). Strategies to help children fall asleep included reading a bedtime story (549 participants, 75.3%) and playing calming music (508 participants, 69.7%) (Table 3).

**Table 3:** Sleep management practices (N=729) Data are presented as frequency (n) and percentages (%).

Practice		Frequency (n)	Percentage (%)
Regular bedtime routine	Always	372	51.0
	Most of the time	221	30.3
	Sometimes	105	14.4
	Rarely	31	4.2
	Never	0	0.0
Child uses electronic devices in the hour before bedtime	Never	79	10.8
	Rarely	183	25.1
	Sometimes	251	34.4
	Often	142	19.5
	Always	74	10.1
Child’s bedtime on school nights	Before 7 PM	37	5.1
	7-8 PM	186	25.5
	8-9 PM	296	40.6
	9-10 PM	178	24.4
	After 10 PM	32	4.4
Child’s wake-up time on school mornings	Before 6 AM	34	4.7
	6-7 AM	261	35.8
	7-8 AM	290	39.8
	8-9 AM	113	15.5
	After 9 AM	31	4.2
Child wakes up during the night	Never	287	39.4
	Rarely (1-2 times a month)	248	34.0
	Sometimes (1-2 times a week)	122	16.7
	Often (3-4 times a week)	54	7.4
	Always (every night)	18	2.5
Child has difficulty falling asleep	Never	283	38.8
	Rarely (1-2 times a month)	257	35.3
	Sometimes (1-2 times a week)	112	15.4
	Often (3-4 times a week)	60	8.2
	Always (every night)	17	2.3
Strategies used to help a child fall asleep	Reading a bedtime story	549	75.3
	Playing calming music	508	69.7
	Allowing screen time	68	9.3
	Using white noise machines	185	25.4
	Giving a warm bath	363	49.8
	Other	104	14.3
Handling bedtime resistance	Strictly enforce bedtime	287	39.4
	Allow the child to stay up longer	68	9.3
	Negotiate with rewards	185	25.4
	Use calming techniques	221	30.3
	Other	37	5.1

Parental concerns and education

Regarding knowledge adequacy, 177 participants (24.3%) felt completely informed, while 259 participants (35.5%) felt mostly informed. Parents obtained information from online resources (514 participants, 70.5%) and pediatricians (399 participants, 54.7%). Interest in further education on sleep hygiene was high, with 539 participants (73.9%) expressing interest (Table 4).

**Table 4:** Parental concerns and education (N=729) Data are presented as frequency (n) and percentages (%).

Question		Frequency (n)	Percentage (%)
Adequate information on promoting good sleep hygiene	Yes, completely	177	24.3
	Mostly	259	35.5
	Somewhat	219	30.0
	Not much	74	10.1
	Not at all	0	0.0
Source of information on children’s sleep hygiene	Pediatrician	399	54.7
	Parenting books/magazines	291	39.9
	Online resources	514	70.5
	Family and friends	222	30.5
	Educational workshops	73	10.0
	Other	36	4.9
Interested in receiving more education on sleep hygiene	Yes	539	73.9
	No	40	5.5
	Maybe	150	20.6

## Discussion

This study aimed to assess parental knowledge and management of sleep hygiene among children in Saudi Arabia, highlighting key practices, knowledge gaps, and implications for child health. The findings provide valuable insights into current parental practices and areas for targeted interventions to improve sleep health in this population.

The study revealed several notable findings regarding parental knowledge and practices related to sleep hygiene. Most parents demonstrated good awareness of the recommended hours of sleep for school-aged children and identified key practices such as limiting electronic device use before bedtime and maintaining a consistent bedtime routine. However, gaps in knowledge were evident, particularly concerning the optimal age for children to stop napping and the effects of caffeine consumption on sleep.

Our findings align with previous studies indicating that parental knowledge significantly influences children’s sleep habits [11]. Similar studies conducted in other regions have reported comparable levels of awareness regarding sleep hygiene practices [11]. However, cultural and environmental factors unique to Saudi Arabia, such as screen time habits and family routines, may contribute to variations in sleep management practices compared to other populations.

The study underscores the importance of targeted educational interventions aimed at improving parental knowledge of sleep hygiene. Effective communication strategies tailored to cultural norms and family dynamics in Saudi Arabia could enhance parental adherence to recommended sleep practices [12]. Healthcare providers, including pediatricians, play a crucial role in disseminating evidence-based information and promoting healthy sleep habits during routine clinical encounters.

Enhancing parental knowledge and practices related to sleep hygiene has significant implications for child health outcomes. Improved sleep hygiene is associated with better cognitive function, emotional regulation, and overall well-being among children [13-15]. Addressing sleep disturbances early in childhood may mitigate long-term health risks and improve quality of life [2].

Limitations

Several limitations should be considered when interpreting the results of this study. The cross-sectional design limits our ability to establish causal relationships between parental knowledge and actual sleep outcomes in children. Self-reported data are subject to recall bias and social desirability bias, which may have influenced participant responses. Additionally, the study sample primarily consisted of parents recruited from pediatric clinics and online forums, which may not fully represent the diversity of parental perspectives across Saudi Arabia.

## Conclusions

In conclusion, this study sheds light on the current status of parental knowledge and practices regarding sleep hygiene among children in Saudi Arabia. While many parents demonstrate awareness of key sleep hygiene practices, such as limiting screen time before bed and maintaining consistent bedtime routines, gaps in knowledge remain concerning the effects of caffeine and optimal napping practices. These findings underscore the importance of targeted educational initiatives aimed at enhancing parental understanding of sleep hygiene, tailored to cultural norms and family dynamics in Saudi Arabia. By improving parental knowledge and implementing evidence-based strategies, healthcare providers can play a pivotal role in promoting healthier sleep habits and ultimately enhancing the overall well-being and developmental outcomes of children in the region.

## Appendices

**Table 5:** Survey questionnaire

Section A: Demographic Information
1. What is your age?
- Under 25
- 25-34
- 35-44
- 45-54
- 55 and above
2. What is your highest level of education?
- Primary education
- Secondary education
- Some college
- Bachelor’s degree
- Master’s degree
- Doctorate or equivalent
3. What is your current employment status?
- Employed full-time
- Employed part-time
- Self-employed
- Unemployed
- Homemaker
- Retired
- Student
4. How many children do you have?
- 1
- 2
- 3
- 4
- 5 or more
5. What is the age range of your child/children?
- 0-2 years
- 3-5 years
- 6-12 years
- 13-18 years
Section B: Knowledge of Sleep Hygiene
6. How many hours of sleep do school-aged children (6-12 years) typically need per night?
- Less than 8 hours
- 8-9 hours
- 10-11 hours
- 12 or more hours
7. Which of the following is NOT recommended for good sleep hygiene in children?
- Regular bedtime
- Watching TV before bed
- Consistent bedtime routine
- Quiet sleep environment
8. What is the ideal time for children to stop using electronic devices before bedtime?
- Immediately before bed
- 30 minutes before bed
- 1 hour before bed
- 2 hours before bed
9. Which of the following is a recommended practice for promoting good sleep in children?
- Allowing caffeine drinks in the evening
- Ensuring the bedroom is dark and quiet
- Allowing irregular bedtimes on weekends
- Using the bed for activities like homework and play
10. At what age should children ideally stop napping during the day?
- Under 2 years
- 2-3 years
- 4-5 years
- Over 5 years
Section C: Sleep Management Practices
11. Do you have a regular bedtime routine for your child?
- Yes, always
- Yes, most of the time
- Sometimes
- Rarely
- Never
12. How often does your child use electronic devices in the hour before bedtime?
- Never
- Rarely
- Sometimes
- Often
- Always
13. What time does your child usually go to bed on school nights?
- Before 7 PM
- 7-8 PM
- 8-9 PM
- 9-10 PM
- After 10 PM
14. What time does your child usually wake up on school mornings?
- Before 6 AM
- 6-7 AM
- 7-8 AM
- 8-9 AM
- After 9 AM
15. How often does your child wake up during the night?
- Never
- Rarely (1-2 times a month)
- Sometimes (1-2 times a week)
- Often (3-4 times a week)
- Always (every night)
16. How often does your child have difficulty falling asleep?
- Never
- Rarely (1-2 times a month)
- Sometimes (1-2 times a week)
- Often (3-4 times a week)
- Always (every night)
17. What strategies do you use to help your child fall asleep?
- Reading a bedtime story
- Playing calming music
- Allowing screen time
- Using white noise machines
- Giving a warm bath
- Other (please specify)
18. How do you handle bedtime resistance or refusal from your child?
- Strictly enforce bedtime
- Allow the child to stay up longer
- Negotiate with rewards
- Use calming techniques (e.g., reading, music)
- Other (please specify)
Section D: Parental Concerns and Education
19. Do you feel you have adequate information on how to promote good sleep hygiene for your child?
- Yes, completely
- Mostly
- Somewhat
- Not much
- Not at all
20. Where do you get most of your information on children’s sleep hygiene?
- Pediatrician
- Parenting books/magazines
- Online resources
- Family and friends
- Educational workshops
- Other (please specify)
21. Would you be interested in receiving more education on sleep hygiene for children?
- Yes
- No
- Maybe
